# Transcutaneous electrical nerve stimulation vs. H-Wave® device stimulation—similar or different?

**DOI:** 10.3389/fpain.2024.1321148

**Published:** 2024-03-19

**Authors:** Ashim Gupta, Stephen M. Norwood

**Affiliations:** ^1^ Future Biologics, Lawrenceville, GA, United States; ^2^ Regenerative Orthopaedics, Noida, India; ^3^ Orthopaedic Surgeon (FAAOS), Austin, TX, United States

**Keywords:** chronic pain, function, electrical stimulation, transcutaneous electrical nerve stimulation, TENS, H-Wave®, H-Wave® device stimulation, HWDS

## 1 Introduction

Chronic musculoskeletal pain, stemming from soft tissue inflammation and injury, has been estimated to occur in up to half of adults worldwide, accounting for hundreds of billions of dollars in healthcare costs (1, 2). Functional deficits including decreased range of motion (ROM) and interference with activities of daily living (ADL) are frequently associated with persistent pain (1, 2). With an increasing prevalence of chronic pain and its associated effects on quality of life (QoL), traditional forms of treatment have included prescription of pharmacological agents including non-steroidal anti-inflammatory drugs and opioids; non-pharmacological approaches like activity modification, physical therapy, cognitive behavioral therapy, complementary and alternative medicine (e.g., acupuncture); as well as various invasive interventions (e.g., radiofrequency ablation) (3). These conventional therapeutics have shortcomings and unwanted side-effects, typically providing only transient symptomatic relief and failing to target the underlying pathology (4). More effective and efficient treatment alternatives having more benign side-effect profiles are sought to specifically target the physiological factors involved in pain generation and transmission.

Over the past decade there has been an increase in utilization of electrotherapies involving differing forms of electrical stimulation (ES), applied alone or as an adjunct to reduce pain and improve function (3). These include, among others, transcutaneous electrical nerve stimulation (TENS) and H-Wave® device stimulation (HWDS). Each of the varying forms of ES have distinct technical parameters, applications and indications related to reduction in pain perception and medication usage, and improvement in function (3). There have been almost no comparative studies distinguishing between these two electrotherapies, or between other forms of ES. While TENS and H-Wave® devices, along with other electrical stimulators, all emit electrical current to human soft tissues, in hopes to mitigate the consequences of various injuries, the technical methods of delivery and resultant clinical implications for each form of electrical stimulation are, in our opinion, clearly distinct.

H-Wave® technology has received 15 separate FDA cleared indications for pain and neuromuscular indications, compared to 2 FDA cleared indications for TENS. The H-Wave® duration of electrical pulse and unusual waveform are so distinctly different from TENS, that it is little wonder that clinical outcomes are not at all similar. Unlike TENS, H-Wave® provides prolonged pain relief and other therapeutic benefits, even after the electrical pulse is stopped (3). These devices operate on a different spectrum, where the two valid technologies have been deployed with completely different design intents (3). TENS provides some short-term pain relief, albeit marginal, while H-Wave® is designed to provide longer-term pain relief, while also enhancing the underlying biological conditions which promote rehabilitation and healing (4, 5).

This brief opinion manuscript focuses specifically on key differentiating factors between TENS and HWDS, in design technology and electrical parameters, U.S. Food and Drug Administration (FDA) clearance for different indications, and mechanism of action and effectiveness based on recent peer reviewed published literature.

## 2 Technology and parameters

TENS devices typically emit a standard high-frequency rectangular (common) waveform with a very short pulse duration, typically around 50 μs (6). H-Wave®, in sharp contrast, uses a proprietary exponentially decaying waveform with very prolonged pulse duration (5,000 μs), up to 100 times longer than TENS, emitting more sustained cumulative soft tissue energy delivery (4, 6).

Conventional TENS, widely utilized, has been described by The International Association for the Study of Pain as high frequency (50–100 Hz), low intensity, with small pulse width/duration (50–200 μs) (7). Less commonly used acupuncture-like TENS is low-frequency (2–4 Hz), high intensity, with longer pulse width/duration (100–400 μs) (7). The pulse amplitude/intensity for TENS is adjustable from 0 to 100 mA (current) peak into 500ohm load (0–50 V, voltage) into each channel (7).

H-Wave® employs a biphasic waveform, with a dual-frequency (2 channels) feature, employed at either ultra-low (2 Hz) or high (60 Hz) frequency (3, 4). At 1,000-ohm load, an H-Wave® device delivers 0–35 mA current and 0–35 V voltage (4).

The technology and parameters for both TENS devices and H-Wave® device are summarized in Table 1.

**Table 1:** FDA cleared indications, technology and parameters, and mechanism of action for transcutaneous electrical nerve stimulation (TENS) and H-Wave®.

Device name	FDA cleared indications	Technology and parameters	Mechanism of action
Transcutaneous electrical nerve stimulation (TENS)	• Symptomatic relief and management of chronic, intractable pain • Adjunctive treatment for post-surgical and post-trauma acute pain	• Rectangular waveform • High frequency (50–100 Hz), low intensity, short pulse duration (50–200 μs) • Acupuncture-like TENS (uncommonly used), low-frequency (2–4 Hz), high intensity, longer pulse duration (100–400 μs) • Pulse amplitude/intensity adjustable from 0 to 100 mA (current) peak into 500ohm load (0–50 V, voltage) into each channel	Pre-clinical TENS studies suggest multifactorial effects: • Peripheral mechanism triggers peripheral opioidergic pathways • Spinal effect attributed to “gate-control theory” reduced inflammation-induced dorsal horn neuron sensitization, altered levels of neurotransmitters including gamma-aminobutyric acid and glycine, and modulation of glial cells • Endogenous analgesia mediated by descending inhibitory activity transmitted via midbrain periaqueductal grey and rostral ventral medulla
H-Wave®	• Chronic pain, post-surgical pain, acute pain, temporary pain • Relaxation of muscle spasm, prevention or retardation of disuse atrophy, increasing local blood circulation, muscle re-education, immediate post-surgical stimulation of calf muscles to prevent venous thrombosis, maintaining or increasing range of motion • Anesthesia in general dentistry, crown, composite and amalgam preparations, periodontal scaling and root planning • Muscle spasms associated with temporomandibular joint (TMJ), and muscle re-education, as in regaining joint control in TMJ	• Proprietary exponentially decaying biphasic waveform • Dual-frequency (2 channels), ultra-low (2 Hz), high (60 Hz), or combined; ultra-long pulse duration (5,000 μs) • At 1,000-ohm load, delivers 0–35 mA current and 0–35 V voltage	Pre-clinical H-Wave® studies indicate that the low frequency (2 Hz) component causes several cumulative physiological effects: • Stimulation of voluntary contraction of smaller, slow twitch skeletal muscle red fibers, resulting in non-fatiguing low-tension contractions • Increased blood flow via vasodilation mediated by nitric oxide, skeletal muscle fiber stimulation, and angiogenesis/neovascularization • Increased rhythmic lymphatic vessel drainage through stimulation of voluntary smooth muscle fiber contractions, causing fluid and protein waste removal from areas of inflammation The high frequency (60 Hz) component results in significant prolonged analgesia, through cumulative, repressive effects on nerve action potentials via sodium channel pump deactivation

## 3 FDA clearance for different indications

TENS devices have been cleared by the FDA for 2 indications, using different FDA 510 K numbers for different manufacturers/distributors, as listed below.

• Symptomatic relief and management of chronic, intractable pain
• Adjunctive treatment for post-surgical and post-trauma acute pain

The H-Wave® device has been cleared by the FDA for 15 specific indications, divided into 4 classifications, as listed below.

• Chronic pain, Post-surgical pain, Acute pain, and Temporary pain
• Relaxation of muscle spasm, Prevention or retardation of disuse atrophy, Increasing local blood circulation, Muscle re-education, Immediate post-surgical stimulation of calf muscles to prevent venous thrombosis, Maintaining or increasing range of motion
• Anesthesia in General Dentistry, Crown, composite and amalgam preparations, Periodontal Scaling and root planning
• Muscle spasms associated with temporomandibular joint (TMJ), and Muscle re-education, as in regaining joint control in TMJ

The FDA clearances for TENS and H-Wave® for various indications are summarized in Table 1.

## 4 Mechanism of action and effectiveness on pain and function

Several pre-clinical studies, summarized in a review by Gibson et al. (5), have shown the mechanism of action of TENS to be multifactorial, likely involving peripheral, spinal, and supraspinal neural mechanisms.

• A peripheral mechanism encompasses participation of peripheral opioidergic pathways
• A spinal effect is attributed to “gate-control theory”, reduced inflammation-induced dorsal horn neuron sensitization, altered levels of neurotransmitters including gamma-aminobutyric acid and glycine, and modulation of glial cells
• Endogenous analgesia is further mediated by descending inhibitory activity transmitted via the midbrain periaqueductal grey and the rostral ventral medulla in the brainstem

Another recent systematic review and meta-analysis by Oliveira et al. (8) assessed the analgesic effect of TENS in pre-clinical animal models. Although meta-analysis demonstrated some pain relief with both low- and high- frequency TENS compared to a control-group, no significant differences were observed between differing frequencies.

One systematic review of TENS clinical trials for chronic pain, reported the quality of evidence to be very low, where TENS seemed to be neither harmful nor beneficial for pain control, disability, health-related QoL, or in decreasing utilization of pain-easing drugs (7). A more recent meta-analysis by Wu et al. demonstrated no improvement in symptoms of lower back pain in patients treated with TENS, although some short-term (<6 weeks) improvement in functional disability was noted (9). Another meta-analysis by Jauregui et al. reported very slight improvement (0.884 on a 0–10 visual analogue scale) in reported pain over the short term (<5 weeks), although there was no such improvement beyond 5 weeks using TENS (10). A 2021 review by the National Institute for Health and Care Excellence (NICE) reported clinically important differences between TENS and sham for pain reduction in two studies, while another demonstrated no difference; no clinically important differences were reported for QoL, physical function, psychological distress, pain interference and pain self-efficacy (11). Johnson et al. in a systematic review and meta-analysis of 381 randomized controlled trials (24,532 participants), reported that pain intensity was somewhat lower during or immediately after TENS application compared to placebo, although other outcome measures were not significantly different to the various comparators; a sub-group analysis comparing application of high vs. low frequency TENS found no significant differences between the two groups (6). Zhu et al. in a systematic review and meta-analysis reported significant reduction in pain and morphine requirement following total knee arthroplasty over a period of 24 h, although no demonstrable reduction was observed at 2 weeks (12). Wu et al. in another systematic review and meta-analysis reported that TENS may significantly reduce pain, improve function and walking ability in patients with knee osteoarthritis, but there was no effect on stiffness (13).

Several H-Wave® pre-clinical studies, recently summarized by Williamson et al. (4), highlighted the mechanism of action of HWDS. The low frequency (2 Hz) component of HWDS leads to several cumulative physiological effects:

• Stimulation of voluntary contraction of smaller, slow twitch skeletal muscle red fibers, resulting in non-fatiguing low-tension contractions
• Increased blood flow via vasodilation mediated by nitric oxide and stimulation of skeletal muscle fibers, and angiogenesis/neovascularization
• Increased rhythmic lymphatic vessel drainage mediated by stimulation of voluntary contraction of smooth muscle fibers of lymphatic vessels resulting in fluid and protein waste removal from areas of inflammation, restoring tissue homeostasis

In contrast, the high frequency (60 Hz) component of HWDS leads to significant analgesia, through cumulative, repressive effects on nerve action potentials via sodium channel pump deactivation, resulting in longer-lasting pain relief (4).

Further clinical systematic review reported significant pain relief and improvement in overall function, along with reduction in pain medication usage post-treatment with HWDS (4). Norwood et al. reported patient-recorded outcome measures (PROMs) for 2,711 non-specific chronic low back pain, sprain, strain HWDS patients, resulting in substantial pain improvement (3.12 on a 0–10 visual analogue scale), with profound positive effects on function and ADL, in addition to benefits like decreased medication use, better sleep, and improved work performance (14). Trinh et al. in a large retrospective cohort study involving end-stage worker's compensation patients, reported no adverse effects associated with HWDS, with significant reduction in pain, opioids/polypharmacy use, and anxiety/depression, while improving overall QoL (2). Williamson et al. in a retrospective cohort study involving first responder firefighters, reported easy device use and no untoward side-effects with HWDS, with statistically significant reduction in reported pain, improvements in ROM and job performance, and increased time spent with family, leading to overall positive health benefits and experience (1).

The mechanism of action for TENS and H-Wave® are summarized in Table 1.

## 5 Discussion

Electrotherapies have evolved as non-invasive alternatives, used alone or as an adjunct in multimodal pain protocols, to circumvent limitations associated with other currently used pharmacological and non-pharmacological treatments for management of musculoskeletal pain and dysfunction (3). This manuscript focuses on differentiating between two commonly used forms of ES, TENS and HWDS, based on technology and electrical parameters, FDA cleared indications, and mechanisms of action and relative effectiveness in reducing pain and improving function.

From a technology and parameters perspective, TENS is capable of emitting either a low or a high frequency component with a relatively small pulse width/duration (3, 6). In contrast, dual H-Wave® device modes allow for either two low, two high, or combined low and high frequency treatments, emitting a much longer pulse width/duration compared to TENS, thereby delivering more sustained cumulative soft tissue energy (4). TENS is FDA cleared for 2 indications, compared to 15 for HWDS. Several recent studies including systematic reviews and meta-analyses have evaluated the efficacy of TENS in ameliorating pain and improving function, with most reporting either no improvement in pain scores or slight improvement only over the short-term (6, 9–11). The majority of TENS studies with decent sample size were classified as either low or very low-quality evidence, showing no improvements in QoL, physical function or psychological parameters (5, 9–14). In contrast, low to moderate quality HWDS studies have consistently demonstrated significant reduction in pain and medication usage, while improving function and overall QoL (1–4, 14).

We conclude that major dissimilarities in technology and electrical parameters, differing clinical FDA clearances, and comparative data from pre-clinical and outcomes from clinical studies clearly demonstrate that TENS and HWDS are distinct forms of ES, each utilizing unique mechanisms of action and having notably different effects on pain and functional outcomes. Further higher quality evidence via prospective, multi-centre, randomized controlled trials with large sample size and longer follow-up are warranted for both TENS and HWDS to further refine their respective abilities to reduce pain and improve function. It should be noted that additional studies are not necessary from a regulatory standpoint, since TENS, HWDS, and other ES devices are classified and/or cleared as Class II medical devices, which have less rigorous research obligations, although ongoing research should still provide valuable insight to patients, providers, and payers.
